# Leadership in Moving Human Groups

**DOI:** 10.1371/journal.pcbi.1003541

**Published:** 2014-04-03

**Authors:** Margarete Boos, Johannes Pritz, Simon Lange, Michael Belz

**Affiliations:** 1 Courant Research Centre Evolution of Social Behaviour, University of Göttingen, Göttingen, Germany; 2 Economics Department & Centre for Statistics, University of Göttingen, Göttingen, Germany; New York University, United States of America

## Abstract

How is movement of individuals coordinated as a group? This is a fundamental question of social behaviour, encompassing phenomena such as bird flocking, fish schooling, and the innumerable activities in human groups that require people to synchronise their actions. We have developed an experimental paradigm, the HoneyComb computer-based multi-client game, to empirically investigate human movement coordination and leadership. Using economic games as a model, we set monetary incentives to motivate players on a virtual playfield to reach goals via players' movements. We asked whether (I) humans coordinate their movements when information is limited to an individual group member's observation of adjacent group member motion, (II) whether an informed group *minority* can lead an uninformed group *majority* to the minority's goal, and if so, (III) how this minority exerts its influence. We showed that in a human group – on the basis of movement alone – a minority can successfully lead a majority. Minorities lead successfully when (a) their members choose similar initial steps towards their goal field and (b) they are among the first in the whole group to make a move. Using our approach, we empirically demonstrate that the rules of swarming behaviour apply to humans. Even complex human behaviour, such as leadership and directed group movement, follow simple rules that are based on visual perception of local movement.

## Author Summary

Our article gives empirical evidence of group coordination mechanisms and basic rules of leadership that assist in leading a human group. Using a computer-based multi-client game that blocks explicit signals or other typical human information transfer, we offer a model of human group movement patterns applicable to group scenarios such as emergency, rescue, and sports where inter-individual communication is hindered but the reading of movement is still possible. Results show that even in these communication-restricted situations, movement of an informed minority that is efficient and consistent can effectively pull the majority towards a target goal.

## Introduction

Schools of fish and flocks of birds move collectively towards a spatial goal [1], [2] despite their large local group sizes and therefore reduced capacity for global or inter-individual communication across the group [3], [4]. Behavioural modelling [5]–[7] and empirical research [8]–[10] have shown that in diverse species, including humans [11], [12], local individual rules are adequate to generate complex collective behaviour at the group level [13]–[15]. There is increasing evidence [9], [16], [17] that not only large swarms but also small heterogeneous groups may be coordinated by local interaction rules.

To explain this “swarming” phenomenon in animals and humans, Couzin et al. [18] created a model in which group locomotion emerges from individuals steering their motion based on the moves of local neighbours. Their model comprises three fundamental parameters described by Aoki [19] and Reynolds [20], stating that members of a swarm (a) become attracted to neighbours' positions within a local range (*cohesion*); (b) align with neighbours' direction and speed within this range (*alignment*) and c) avoid neighbours within a predefined radius (*collision avoidance*). In order to incorporate the influence of those individuals with information about a preferred goal, a weighted direction vector was added [18] to investigate the dilemma of informed individuals pursuing their preferred goal while trying to remain with the group [21]. In their computer simulation model, Couzin et al. [18] deduced that a proportionally small number of directionally informed individuals can channel the naïve (uninformed) members of the swarm to the target of the directionally informed. Neither the informed nor the naïve need to recognise each other, be aware of the informational gap, or practise active signalling. Assumptions about inherent personal distinctions (e.g. personality traits or social cues) need not to be present in order to explain effective movement leadership.

The purpose of the present study was to test whether such “swarm-like” human movement and leadership behaviour empirically holds for a small group of humans restricted to ‘reading/transmitting’ only movement behaviour. To do so, we developed the computer-based HoneyComb multi-client game as our investigative platform. The elements of this virtual game were designed to eliminate all sensory/communication channels except the perception of player-assigned avatar movements on the playfield. To create experimental factors of individual motivation towards the two swarming behaviours – “cohesion” and “alignment” – without experimenter's direct behavioural instructions, we implemented within-group graded monetary incentives, an informed minority of players with a higher-rewarded target, and an uninformed majority of players with lower but equal reward targets.

This experimental paradigm differs substantially from the approach taken in three other studies on human movement and leadership by Dyer et al. [11], [22] and Faria et al. [23], where the minority leadership prediction of the Couzin et al. model [18] was investigated in a face-to-face group situation. All three studies conducted live experiments of humans walking in a circular arena with a landmark randomly assigned as a target to the informed individuals. This naturalistic approach has benefits in terms of *ecological* validity. But these studies fall short in discretising the spectrum of human communication: defining where communication among humans begins in the phenomenon of leading human group movement. Firstly, face-to-face procedures, despite instructions not to communicate, could not preclude the transmission of non-verbal cues between participants (e.g. eye contact, facial expressions, shared/non-shared social categories of age and gender). These cues likely functioned to signal intention towards the goal or to unite participants to “flock” based on the perceived affiliation to the same social category, almost certainly confounding results. Second, instruction methodology in these studies was direct, which is incongruent with the above-mentioned swarming conditions as “local” rules. In the three studies' standard set of instructions, the model [18] parameter “group cohesion” was directly translated into the investigator's explicit instruction to participants to “remain as a group of eight (ten resp.)”. “Collision avoidance” was implemented by the instruction “to stay within an arm's length of another individual” [11]. Our platform ensures *internal* validity because the study design of humans moving their avatars on a virtual playfield eliminates all sensory/communication channels between the human participants other than observed directional movement. By using monetary incentives we introduce a motivational factor for group movement but avoid any direct instruction; therefore, any potential leadership influence of movement remains with the transmitting/reading of participants' movements rather than external or internal socio-cognitive sources.

The goals of our study are to address three fundamental questions regarding basic human coordination mechanisms and the emergence of patterns of group leadership in the complete absence of communication mechanisms and pre-knowledge of information differentiation other than the perceived movement of others: (I) Can humans coordinate their avatars' movement under such extremely restricted communication conditions? (IIa) Can the informed minority lead the uninformed majority to their goal field, (IIb) even with the additional restriction for each group member to perceive only local movement relative to his/her proximity? (III) Which movement behaviour of the minority is the best predictor for the success of leading the majority to a target?

## Results

### New experimental paradigm: Virtual HoneyComb game

**Figure 1:**
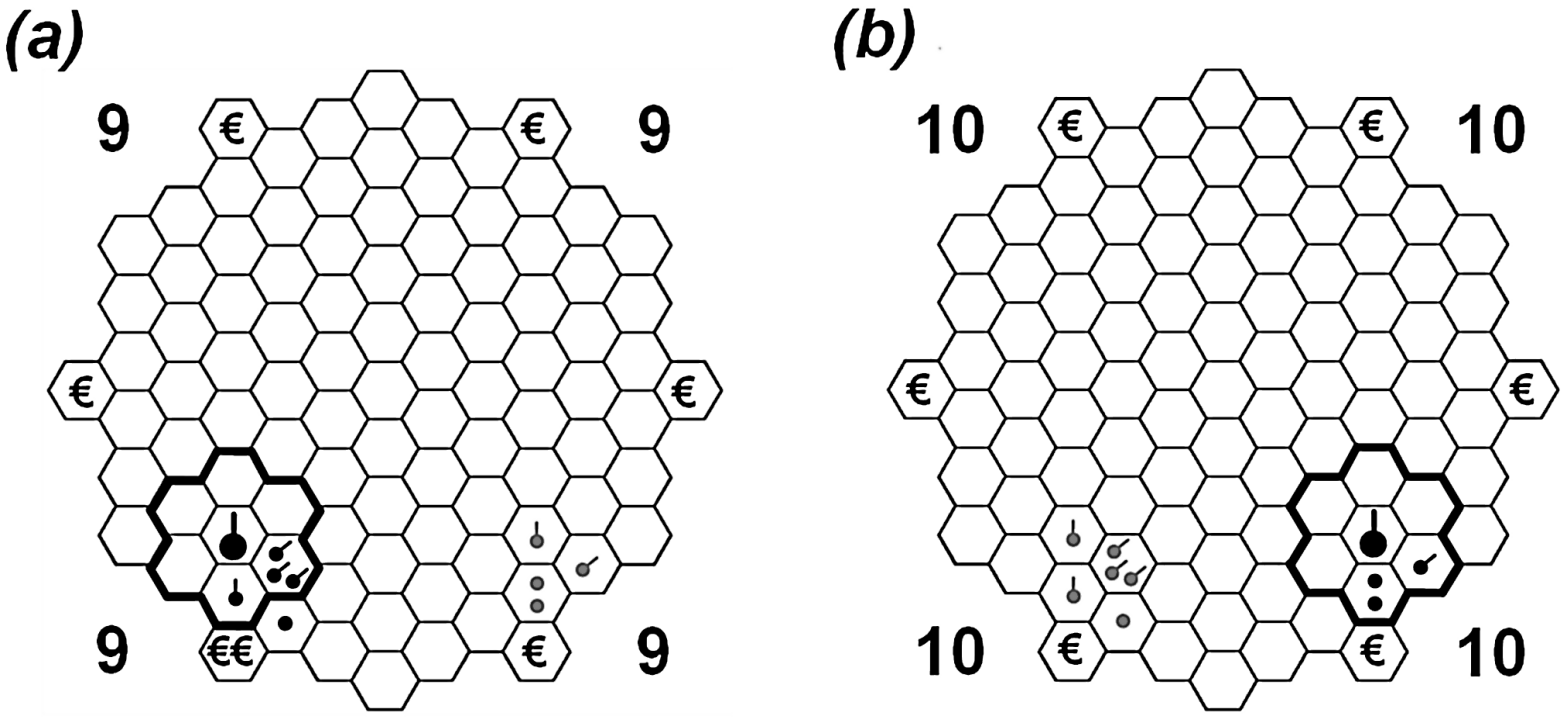
The HoneyComb playfield. The screenshots show two different views of the same virtual playfield composed of 97 hexagons, one from the perspective of an *informed* player *(a)*, the other from the perspective of an *uninformed* player *(b)*. In this example, the pertinent players (represented as larger dots) have nearly reached their goal fields in the lower left (*a*) and lower right (*b*). Whereas the ‘€’ money depots are visible to uninformed as well as informed players, the ‘€€’ money depot (randomly placed on one of the ‘€’ money depots) can only be seen by informed players *(a)*. Uninformed players perceive the ‘€€’ money depot only as a regular ‘€’ money depot *(b)*. The numbers in the four corners of the playfield indicate to the pertinent player how many moves out of 15 are still available; in this case, 9 *(a)*, and 10 *(b)*. In the experimental condition of *local* perception, the focused player sees only the dots in the area inside the black contour, whereas other dots – here marked in grey – are invisible to him/her. In our screenshot, for the informed player *(a)* four group members' black dot avatars are inside the perception radius and thus visible; for the uninformed player *(b) three* group members are in visual range. In the experimental condition of *global* perception, everything is visible to the players. See also supporting information for video files from one experimental group (Video S1 for uninformed and Video S2 for both – uninformed (right side) and informed (left side) – perspectives).

A total of 400 subjects participated in our experimental application of the HoneyComb game, conducted in a quiet zone of the main lecture building of a large university in Germany (see *Material and Methods* for details on *Pre-test*, *Participants*, *Experimental Procedure*, *Locomotion*, and *Ethics Statement*). In the HoneyComb game, participants interacted anonymously in groups of ten players by means of computers connected via a local network. Study question (I) – can humans coordinate their avatars' movement under such extremely restricted communication conditions – required absolute exclusion of any sort of communication other than observed directional movement. Each player was surrounded by computer-station partitions and was required to use ear plugs to prevent verbal and nonverbal communication during the experiment. On the virtual playfield resembling a honey comb of 97 hexagonal spatial fields, each participant was represented as an avatar, i.e. as a black dot identifiable only to him/her by being twice the size of the other nine co-participants' dots (Fig. 1). At the beginning of the game, all players' dots were positioned in the centre of the honey comb. In each of the 15 available moves, the players could navigate their dot via mouse-click to one of six neighbouring fields from their respective point of departure. An incentive structure operationalising the model parameters was implemented via six spatial goal fields rendering monetary payoffs (€ or €€) (*alignment*). If a player arrived at a payoff field, his or her payoff would be multiplied by the number of co-players' avatars standing on this payoff field at the end of the game (*cohesion*). To gain a high payoff, coordinated choices were thus advantageous. The criterion for ending the game was either that all players' avatars stood on payoff fields, and/or that all players had used all available moves.

To explore study question (IIa) where we tested the first experimental factor regarding the informed minority being able to lead the uninformed majority to their goal field, a minority/majority information differentiation within each of the ten-person groups was established: a minority of two randomly selected players was informed of the location of their one highly-rewarded €€-goal field in addition to five lower-rewarded €-fields (Fig. 1a). A majority of eight players were notified of six equally lower-rewarded goal fields (Fig. 1b). Neither the highly-rewarded “informed” nor the lower-rewarded “uninformed” knew whether they were in the majority or in the minority or that there was a reward difference among players.

To address question (IIb) where we assessed whether locomotive coordination and leadership would be possible only within *global* or also within *local* perception radius of the players, the second experimental factor was implemented where members were additionally restricted to be able to perceive only local movement relative to his/her proximity. The 40 ten-person groups were randomly allotted to either the *local condition* (*n* = 20 groups) limiting the participants' sight to events only on the neighbouring fields of their dot's position or to the *global condition* (*n* = 20 groups) disclosing an overview of all events on the playfield.

It is important to note that the higher incentive of the informed players potentially creating leaders does not necessarily generate follower behaviour; it simply establishes a motivation factor normally present in groups where leaders and followers emerge. Both behaviours – leading and following – are necessary for effective leadership to occur [24]. That the majority, i.e. a higher than probabilistically-expected number of non-informed group members, will follow said informed individuals is not guaranteed by the mere presence of a motivation factor as it is yet to be seen whether its presence invokes any sort of group-member leading and following behaviour.

If leadership of the informed minority is indeed observed, it then piques the question of which characteristic(s) of their movement behaviour makes those minority members successful in leading the uninformed majority members to their goal field. In the choice of variables characterising the informed players' movement behaviour most apt to successfully lead the majority to the minority's preferred field (question III), we focused on the initial stage of the game, as these behavioural variables (effect of local-only or globally-perceived movement by the minority (informed) and majority (uniformed) members; whether and to what extent being the first minority leader to move has an effect on follower behaviour; and effect of path consistency among the minority leaders) have a greater claim for being influential and thus allowing for a (tentative) causal interpretation of our results.

This section describes the variables coded to test our hypotheses regarding the relationship between the visibility radius of the players, the initial moves of the informed players and their leadership success, which is measured by the *number of arrivals of uninformed players* on the informed players' preferred outcome field.

#### Local vs. global perception

We coded a binary variable *local* equal to unity if the local-condition was active, i.e. a value of 1 if the players' visual perception was limited only to fields bordering the players' current position (see Fig. 1); a value of zero was coded if global perception was permitted. The *local*-condition was imposed by the experimenters. Being able to perceive other players only within a limited area around one's own position (study question IIb) might have aided potential leaders as it potentially provided players with an incentive to keep their avatars together as a group. On the other hand, limited perception posed a real threat to group coherence because players could lose sight of each other's avatars. Hence, the effect of this condition was a priori unknown.

#### First-mover behaviour

We coded a binary variable *first* with the value of 1 if one of the informed players was the first player to move. As payoffs for all players depended on successful coordination towards a goal field, all players had an initial incentive to follow the lead of the first-mover. Thus, individuals setting an example by being the first to move from their start position were predicted to incur a following and thus invoke effective leadership. And, as the informed individuals emerged as those with the highest “*energetic requirements*” [24], they were predicted to initiate movement activity and lead the group [25]–[27]; in other words, people with enough motivated energy to not be inhibited by inertia or lack of information were predicted as first movers. Leadership by a single individual or a minority would thus emerge as a consequence of informational heterogeneity within the group [28].

#### Path similarity and direction

Finally, we quantified the degree of similarity between the initial moves of the two informed minority players. Minority influence – as is empirically confirmed by a vast number of studies in social psychology based on Moscovici's seminal work [29] – is more likely to occur if the behaviour of the minority is consistent. Exposing similar behaviour within the minority will increase the appeal to the majority, leading to a higher chance of following behaviour. We thus coded two mutually exclusive binary variables: *same* or *direction*. If the informed players decided to take the shortest route towards their preferred field, they had two initial options (see Fig. 1). The variable *same* took on the value 1 if both informed players chose the same field with their first move and this field was one of the fields lying in the direction of their preferred goal field. The variable *direction* was allotted 1 if both players moved in their preferred direction but chose different fields with their first move. As both events were predicted to provide a clear signal to uninformed players, we expected to find a positive association between *same* and *direction* and the number of arrivals, with a larger effect for *same*. The variable where at least one of the informed players did not move into the preferred direction initially was omitted from the analysis.

#### Personality and control variables

Using a post-participation questionnaire, Big Five *personality variables* of the players (openness to experience, conscientiousness, extraversion, agreeableness, neuroticism [30]), as well as *agency and communion* [31], were measured. In order to control for effects of their movement behaviour taken as a whole, the degree of *path similarity* between the locomotive paths of the two informed minority players, their mean *path length* to their goal field, their *mean latency of movement*, and their mean *starting order* were measured and tested for effects on the arrival rate of the uninformed players on the ‘€€’ money depot. Potential impact of computer skills was controlled by using the *computer literacy* [32] scale included in the post-participation survey.

### Successful group coordination in the absence of communication (I and IIa)

For all players, only the money depots had a subjective value. We therefore expected that 0% players would arrive elsewhere in the hexagon. For the uninformed majority, the one ‘€€’ money depot (unknown to them) was expected to be as attractive as each of the five ‘€’ money depots, leading to a predicted probability of a sixth (16.67%; *n* = 53) of the uninformed majority to arrive at the ‘€€’ money depot by the end of the game. However, empirically, 34.37% (*n* = 110) of uninformed players arrived at the ‘€€’ money depot. In order to test the null hypothesis that the arrival rate on the ‘€€’ money depot was less or equal to a sixth, we implemented a two-stage bootstrap procedure that accounted for the hierarchical structure of the data: 1,000 bootstrap estimates of the arrival rate were obtained by first sampling 40 groups with replacement before sampling eight players within each group with replacement. Our result indicated that the arrival rate was significantly higher than a sixth (P<0.001). This means that informed minority players, even in the absence of verbal and non-verbal communication, were able to significantly influence their uninformed majority co-players to move their avatars to their ‘€€’ target, primarily because informed minority players initiated movement earlier than uninformed majority players: in 72.5% of all observed groups, informed minority players showed a mean starting rank less than the middle starting rank of 5.50 for their initial move (binomial test: *P*<.001).

### Effective leadership of the informed minority under global vs. local perception (IIb)

**Figure 2:**
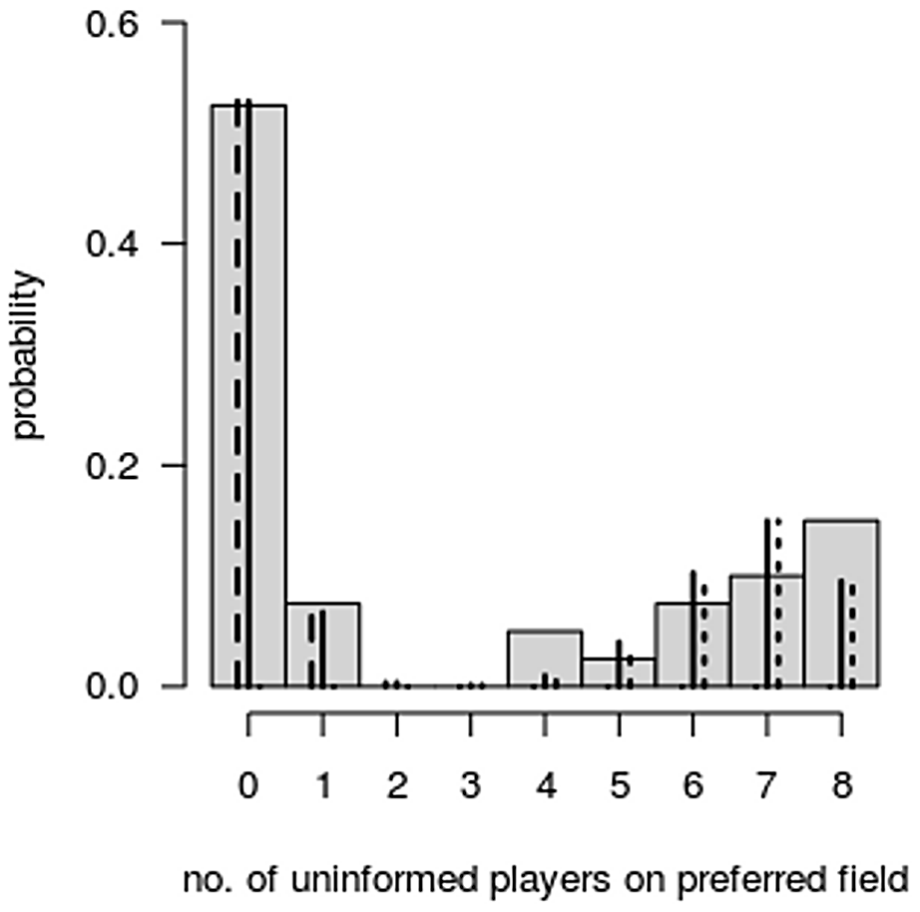
Histogram of arrivals (n = 40), fitted mixture of two binomials (solid lines), and contribution to probability mass function of the two binomials (dashed and dotted lines, respectively).

*Fig. 2* shows a histogram of the number of arrivals of non-informed players' avatars on the informed players' preferred outcome field (*arrivals*) for the 40 groups (grey bars). In principal, *arrivals* might take on all discrete values between zero and eight. Hence, a starting point for modelling the distribution of the variable *arrivals* would have been a binomial distribution with eight trials. However, from visual as well as statistical inspection of the univariate distribution, we stipulated a finite mixture model entailing two binomials with success probabilities π_1_ and π_2_ and mixing parameter α. Parameters were estimated via maximum likelihood methods (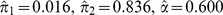). The probability mass function of this mixture model and the contributions of the two components are depicted in *Fig. 2* (dashed lines for the first component, dotted lines for the second). We successfully tested the null of one component against the alternative of two components following the parametric bootstrap procedure proposed by McLachlan [33] (*P*<.001) – in this case, by calculating the likelihood-ratio test statistic for 200 bootstrap samples from the null model.

### Initial locomotive pattern of the minority (III)

In a second step, we introduced covariates to the model by letting the mixture parameter α depend on a linear combination of variables describing the initial movement behaviour of the informed minority.

Descriptive statistics for all variables and associated hypotheses are summarised in *Table 1*. Negative coefficients signal positive associations between *arrivals* and the respective variables in the model we specify, as we explain in the next section.

**Table 1:** Summary statistics and hypotheses.

*Conditions*	Mean	Std. deviation	Hypothesis
*arrivals*	2.75	3.38	–
*local* a	0.50	–	H1: 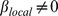
*first*	0.28	–	H1: 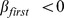
*same*	0.20	–	H1: 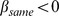
*direction*	0.60	–	H1: 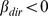

*Note*.

a : experimental condition.

As mentioned above, covariates affect the outcome variable through the mixing distribution. Hence, the distribution of 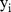, the number of arrivals in game *i*, given a vector of covariates 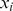, is calculated by
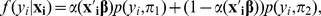
where 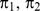 and the 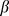
s are parameters to be estimated; 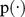 is the probability mass function of a binomial distribution with eight trials; 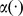 is the logit function; and 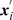, the matrix of covariates, includes a constant. Note that for 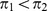 (which happens to be the case in our application), a positive parameter value signals a negative association between the number of arrivals 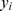 and the variable of interest.

Results of our statistical analyses are summarised in *Table 2*. In addition to parameter estimates and asymptotic z-values for the 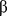
s, we report the number of parameters (*p*), the retained minimum of the negative of the log-likelihood (*-l*), and the Akaike and Bayes model selection criteria (*AIC* and *BIC*, respectively). The first column reports results from an *“empty”* model, i.e. the model without any covariates described above where only the mixing parameter 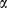 is transformed to a constant via the logit link. Despite being non-informative in terms of the determinants of the number of arrivals, this three-parameter model was the most parsimonious and already yielded a reasonable fit. It therefore served as a useful comparison.

**Table 2:** Parameter estimates for mixtures of two binomials (see text), negative log-likelihood (-l*), number of parameters (p), and model selection criteria (AIC and BIC) (N = 40 groups).

	(1)	(2)	(3)	(4)	(5)
π_1_	0.016	0.016	0.016	0.016	0.016
π_2_	0.836	0.836	0.836	0.836	0.836
β_0_	0.405	0.619	0.493	0.251	16.431***
	(0.131)	(0.201)	−0.159	−0.088	(6.444)
β_local_		−0.448	–	0.560	–
		(−0.188)		(0.273)	
β_first_	–	–	−0.310	31.166***	–
			(−0.188)	(26.058)	
β_first×local_	–	–	–	−32.894***	–
				(−27.519)	
β_same_	–	–	–	–	−18.377***
					(−19.645)
β_dir_	–	–	–	–	−15.920***
					(−6.713)
-l*	62.43	62.22	62.34	58.69	54.50
p	3	4	4	6	5
AIC	130.86	132.44	132.67	129.37	118.80
BIC	135.93	139.20	139.43	139.51	127.25

*Note*: Asymptotic *z*-values in parentheses.

*** and * denote significance at the one-, and ten percent level, respectively.

As described above, we also measured different variables describing the overall locomotive behaviour of the informed players. Incorporating these variables in our empirical model, we found that, while sometimes significant, these specifications generally resulted in inferior model fits. Informed decisions taken during the very early stage of the game led to very distinct outcomes, as confirmed by the distinctly and visibly bimodal distribution of the dependent variable *“arrivals of the uninformed players”* (see *Fig. 2*).

The second column reports results from a model variant in which we tested for the effect of the local-condition. We found a mildly negative coefficient on the local-dummy. That means that constraining the perception of the players tended to increase the number of arrivals on the informed players' preferred field. However, the coefficient is insignificant at conventional levels. In column three we tested whether the success probability increased when one of the informed players was the first to move. The coefficient does have the expected negative sign but is also insignificant. To further investigate these tendencies, we interacted the variable *first* with *local*. Demonstrating decisiveness by making the first move might have been more important when there was a real threat of losing sight of other players. This is indeed what we found, as demonstrated by the significant coefficients in column four on *first* and the *first×local* interaction term.

In column five, we tested whether moving into the same direction increased the success probability. We found that both variables, *same* and *direction*, exerted a positive effect on the number of arrivals, with a quantitatively larger effect for the former. We conclude that a condition of informed minority players moving in cohesive lockstep strongly predicts the outcome of the game.

In addition, the model variant reported in column five was the only model variant that performed superior in terms of both model selection criteria when compared to the non-informative model variant in column one.

**Figure 3:**
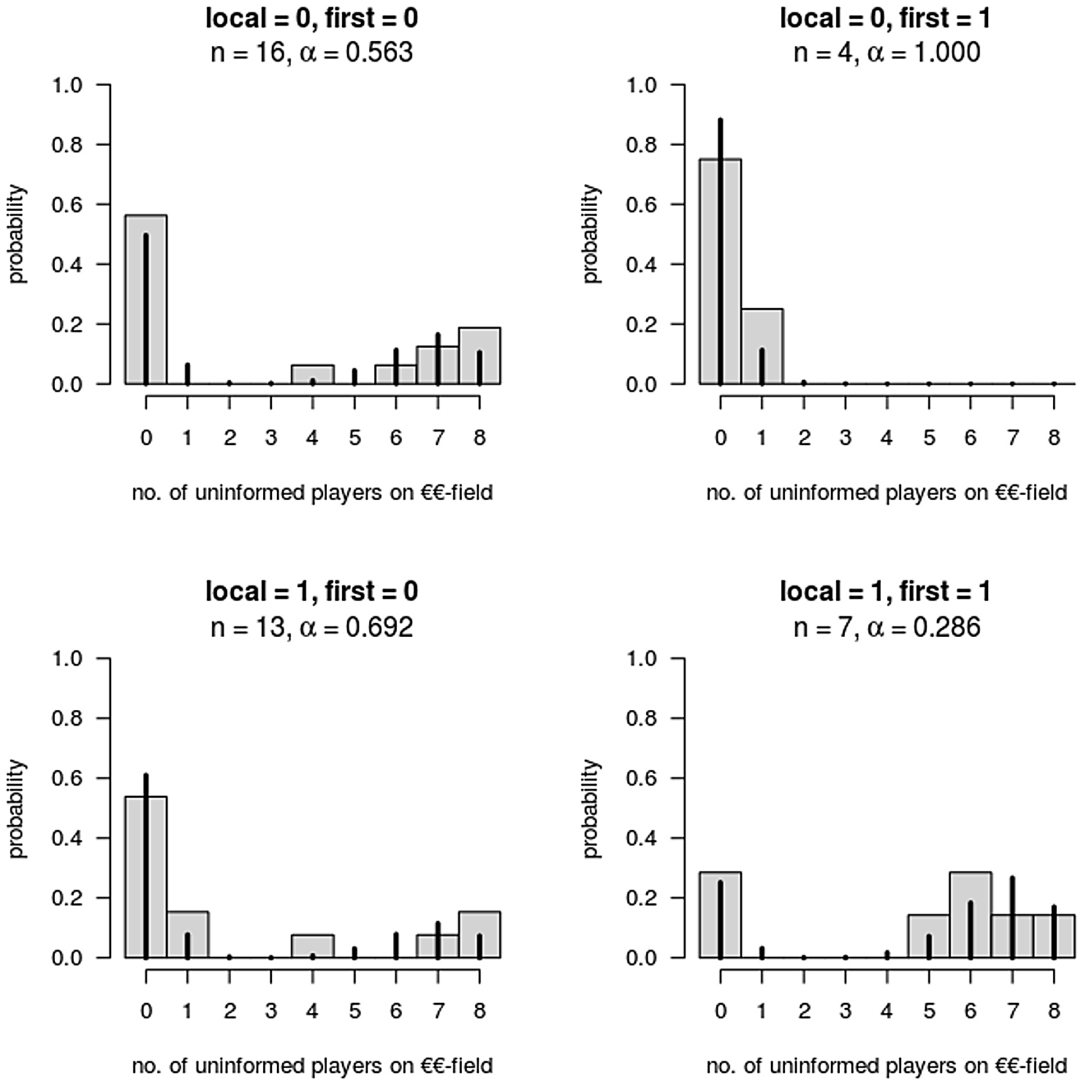
Histogram of arrivals and fitted model (solid lines) under combinations of conditions local and first (n = 40).

**Figure 4:**
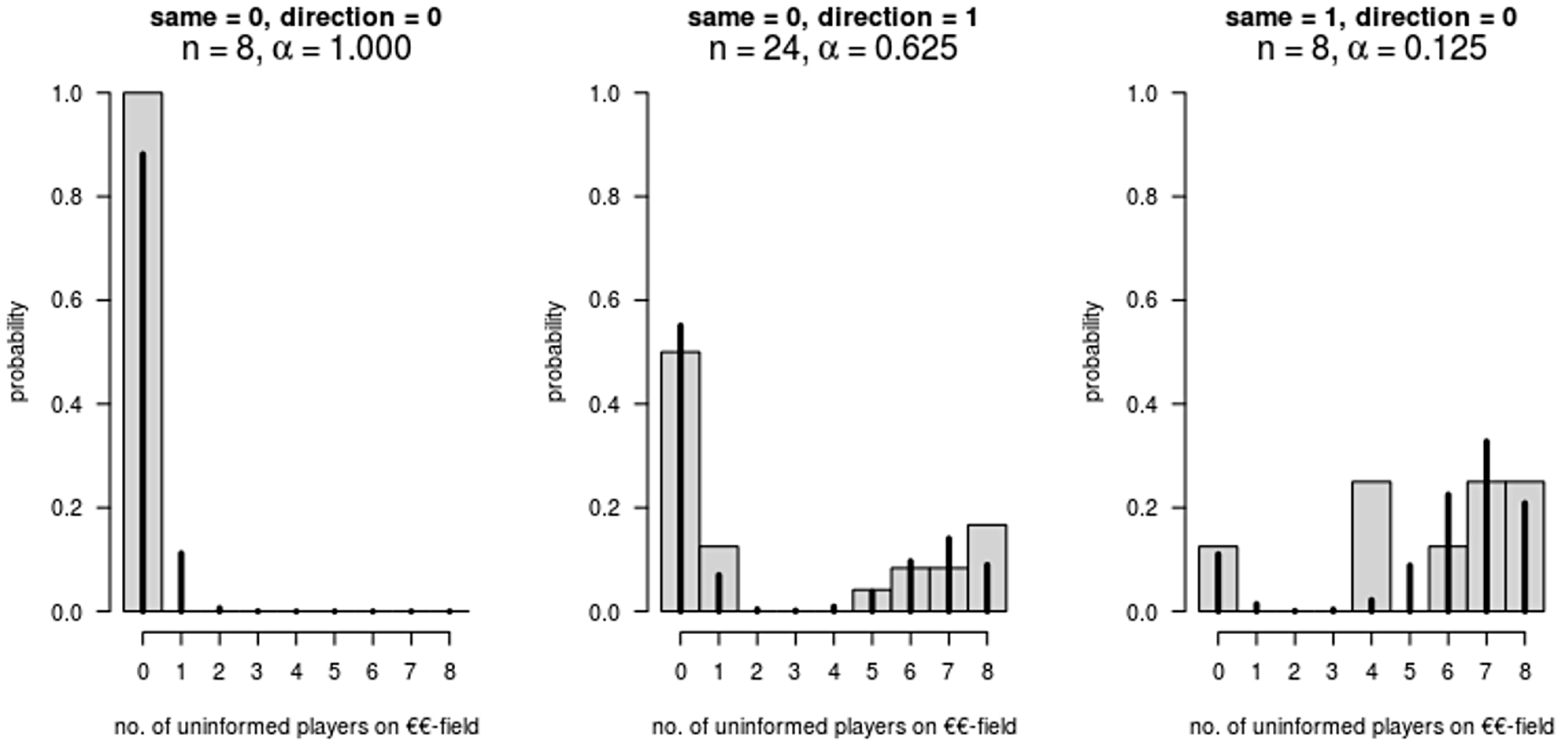
Histogram of arrivals and fitted model (solid lines) under conditions *same*, *direction*, and *omitted* category (*n* = 40).

*Fig. 3* and *4* depict histograms of the arrivals under conditions specified in columns 4 and 5 of *Table 2*, respectively, against the fitted model variants. Making the first move was associated with a lower number of arrivals if players disposed of a global overview of the playfield. Only in 4 out of 20 games was the player able to make the first move as one of the informed. If the limiting local-condition view of adjacent players only was active, the predictor *first* was associated with an increasing success probability, as can be seen from the lower two panels *in* *Fig. 3*.

The fit of the model variant in column 5 is demonstrated in *Fig. 4*. Note that in the eight games in which the informed players did not move their avatars into the direction of the preferred field, the number of arrivals was always zero. Hence, the second distribution received almost zero weight. This predicted number of arrivals changed drastically when the two informed minority players demonstrated cohesion and thus provided other players with a signal about their preferred direction.

## Discussion

We confirmed that even in humans, self-organised patterns of collective movement emerge without assuming global information, strategic or other cognitive complex considerations, or any other sort of communication besides transmitting/reading one's own and other participants' movement behaviour. We could predict the most effective locomotive behaviour of an informed minority to pull an uninformed majority's avatar movements to a specific goal field (i.e. invoking effective leadership) when all channels of communication were reduced to pure movement on a virtual playfield, even when perception radius limited transmitting/reading movement information to/from local neighbours [3], [4], [34]. The successful locomotive pattern of the informed minority players was achieved by moving their avatars analogously into the same direction, and – when perception radius was restricted to only neighbouring fields – making use of an initial mover's effect [28]. The results show that the immediacy of the very first move of the informed minority players and consistency of their initial move – compared to their overall locomotive pattern, path similarity, path length, movement latency, and starting order – are the most effective behaviours for influencing the majority to follow. Personality traits and computer literacy of informed minority players were not crucial to their success in pulling the uninformed majority's avatars to their preferred goal field.

Our results corroborate one of the core assumptions of Couzin et al.'s [18] modelling of the synoptic process of individuals coordinating via movement as a channel of information transfer. This empirical evidence that the ability exists to coordinate behaviour via the transmitting/reading of movement alone might have high adaptive value in situations where human groups experience restricted communication and therefore are forced to lead solely via movement trends of immediacy and consistency. Applied to emergency, rescue, and sport scenarios where face-to-face communication is hindered but movement is still possible, the group success rate towards a desired goal could be maximised via movement initiation that is decisive: i.e. consistent, immediate and therefore consequent. For instance, leadership personnel of such groups could be trained accordingly in using simple behavioural mechanisms for leading masses of uninformed people to emergency exits or secure areas.

But the question here is whether our results – obtained in a virtual environment where movement was performed by representations of the group participants as black dots on a virtual playfield – are applicable to these actual (vs. avatar) human movement scenarios. It can be argued that the representation of the human participants in the rather abstract avatars of black dots in a virtual environment exacerbated the lack of sense of connection between the participants [35]. Because our avatars were designed to reduce social meanings and social roles to a minimum, coupled with the work by others showing avatars mediate the body in virtual settings [36] and that anthropomorphic as well as polymorphic representations in a virtual environment facilitate feelings of embodiment [37], we felt confident that human behavioural association would not be diminished by not transmitting influences of body shape, gender, age and other hierarchical standings [38] by our HoneyComb avatars. Also, the physical presence of their co-players sitting at tables besides them – although behind partitions and with earplugs – likely confirmed feelings of human embodiment. Although we did not ask participants whether they felt sufficiently represented by their avatar and also perceived their co-players as humans moving on a field in this study, the high percentage of participants reporting high levels of embodiment in a subsequent, yet to be published study might hold as an additional argument.

The use of movement as a basic signal to maintain group cohesion and indicate direction appears to be an innate behaviour that does not require complex cognition [39], [40]. As in models describing collective pedestrian behaviour as “spatiotemporal patterns” emergent “through the nonlinear interactions of pedestrians” [12, p. 368], we could empirically show that collective group movement and leadership – in other words non-random behaviour – emerged empirically from implementing the assumed parameters of the swarm behaviour models [18]–[20] into incentives within our HoneyComb virtual movement game.

Unlike studies on pedestrian behaviour [16], [17], [41], we did not base our approach on the sociological concept of a group allowing social interaction and social ties between individuals. Nevertheless, fundamental human mechanisms of collective movement as trails, unidirectional lanes or a basic principle of least effort [7] identified by these researchers are likely to underlie the movement patterns identified in our study.

To explain such complex collective phenomena in humans, we needed neither to assume humans communicating with each other, nor to apply other higher order cognitive and/or social competence nor mutual acknowledging of intentional behaviour in leading (and being led) as none of the players knew that there was any informational difference in the group. Methodologically, this means that functional behavioural complexity at the group level does not necessarily equate with an underlying cognitive complexity at the individual level, but can also be explained by distributed embodied cognition [39], local heuristics [42] or even the principle of least effort [7].

Due to the physical restrictions of our study setup, as compared to the Dyer et al. experiment setting [11], [22], [23], it is highly unlikely that players were able to signal each other their intention, cognitive or motivational state. The rationale behind our study's strident physical restrictions was to focus on mere movement behaviour as a type of inadvertent social cue or – even less – the possibility that the players do not apply mutual responsiveness but in the simplest case practise spatial pattern recognition [39], suggesting the most reductionist explanation possible for effective leadership behaviour in human groups.

Methodologically, this study's application of the HoneyComb paradigm makes heuristic use of existing formal models of swarming behaviour [18]–[20] in order to (a) implement the model parameters into experimentally set behavioural incentives to participants, (b) test whether their empirical behaviour fits specific model predictions in this study of leadership of a minority over a majority, and (c) describe patterns of this observed behaviour. With step (c) we reach beyond the existing formal models we built upon.

The next investigations will likely entail additional applications of the HoneyComb computer-based multi-client game approach to analyse the process patterns on a more sophisticated level, i.e. Markov chains and topological models, and design and execute further experiments in order to identify additional influencing factors of leadership (e.g. initial positioning, visual appearance, communication, and the identifiability of informed minorities). Along the lines of the experimental studies on schooling fish by Couzin et al. [43] and Tunstrøm et al. [44], we could gain deeper insights into the mutual dependence of leadership and followership by manipulating size as well as informational status of the subpopulations of a group and of the size of the group as a whole. We have already run further HoneyComb paradigm based experiments where we manipulated the colours of the avatars in order to investigate how the distinction of “minimal groups” [45] of similar individuals can influence their flocking behaviour and their mutual perception as well as their identification with “their” group. Another application of the paradigm has been to install two minorities with opposing goal fields to investigate how leadership and group fission and fusion work under these conditions.

In sum, our approach and the results of this study provide a new paradigm on boundaries of communication in the influence of coordinated human movement that could readily be extended to additional questions regarding consensus and leadership dynamics.

## Materials and Methods

### Ethics statement

Data collection and data analysis procedures in this project “Leadership in coordination games” have been approved by the Ethics Committee of the Georg-Elias-Müller Institute for Psychology of the University of Göttingen (proposal 039/2012).

### Pre-test

The impact of cohesion incentives independent of other factors was tested in five ten-person groups. All groups consisted only of uninformed players with six equal reward targets (‘€’ money depots). The five groups reached a mean arrival rate of 92% on one single ‘€’ money depot at a time (*s.d.* = 8.37%, minimum of 80%, maximum of 100%).

### Main study

#### Participants

A total of 400 students from 42 different majors participated in the experiment (17.8% business studies, 14.3% jurisprudence, 9.3% economics, and 58.6% other; mean age 24.49 years; *s.d.* = 3.63; 41.1% female).

#### Experimental procedure

Participants in a computerised experiment platform (HoneyComb game) were randomly recruited as 40 ten-person groups. The computer server randomly chose two informed players (minority), leaving eight uninformed players (majority); “informed” meant that the location of the high-reward depot was indicated on their HoneyComb game playfield. In a second step, these groups were randomly assigned to either the *local* or to the *global condition* (*n* = 20 groups per condition). Informed as well as uninformed individuals did not know that there were any informational differences. After completing the game, participants filled out a questionnaire for the assessment of personality traits, computer literacy and demographic data (see Data Set S1 and codebook Table S1). Finally, participants were anonymously paid via neutral envelopes containing their individual rewards and fully debriefed afterwards.

#### Locomotion

Each participant controlled a black dot on a 97-hexagons playfield with a mouse. This dot, which was twice the size of the co-players' dots, could be moved to each of the six neighbouring hexagons by left-clicking. When participants moved their cursor to a hexagon, a framework of these possible fields appeared during mouse roll-over. After participants had made a move, the cursor was transformed into an hourglass for 1500 ms, during which time period no further moves were possible. After each move, a small tail was shown for each player, pointing in the direction from which he or she hailed (see Fig. 1). These tails disappeared after 4000 ms if players chose to delay their next move accordingly. All visual components were displayed in black, white, and shades of grey to control for participants who may have been colour blind. Prior to the game, participants were given instructions to read through with simplified illustrations of the playfield.

An archive version of the software which was used for the experiment (Software S1) and a technical manual how to use it (Text S3) are available as supporting information. More information about current developments of the HoneyComb project is given under https://www.psych.uni-goettingen.de/en/communication/research/honeycomb-c-coordination-in-human-groups.

## Supporting Information

Data Set S1 Data set of the current experiment.
(XLS)

Software S1 Archive version of the software which was used for the experiment.
(ZIP)

Still Video S1 HoneyComb playfield from the perspective of an uninformed player.
(TIF)

Still Video S2 HoneyComb playfield from perspectives of an uninformed (right side) and an informed player (left side).
(TIF)

Table S1 Codebook of the current experiment's data set.
(XLS)

Text S1 Legend to Video S1.
(DOCX)

Text S2 Legend to Video S2.
(DOCX)

Text S3 Technical manual how to use the HoneyComb software.
(PDF)

Video S1 Example of collective movement from the perspective of an uninformed player.
(MOV)

Video S2 Example of collective movement from the same group – from perspectives of an uninformed (right side) and an informed player (left side).
(MOV)
