# Supplementary material for: Leadership in Moving Human Groups
Source: PLoS Comput Biol. 2014 Apr 3;10(4):e1003541. doi: 10.1371/journal.pcbi.1003541 (PMC3974633; doi:10.1371/journal.pcbi.1003541)
Supplement: Software S1 — Archive version of the software which was used for the experiment. (ZIP) [file pcbi.1003541.s002.zip › HC_Guide.pdf]

GEORG-AUGUST-UNIVERSITÄT
GÖTTINGEN

The HoneyComb© Software

1 Getting started

First check whether a java run time environment is available on your computer. Open a console (in Windows you get one by typing cmd after clicking the start button). Try the command

```
java   -version
```

May be you need to install java before you can continue.

1.1 Running the demo on a single screen

When you have unzipped `HC.zip` into a folder, no config-files are present in the base folder, so that the programs will use default values. Start

`HC-demo.bat` (windows) or `HC-demo.sh` (Linux)

by clicking on it in your file-browser. In Mac OS X open a terminal (spotlight > search > terminal), change directory to your HC-folder and type

`./HC-demo.sh ENTER`.

The demo runs with two clients and one control panel on a single computer and a single screen.

If that doesn't work for you, open three terminals, in each go to the folder to where you have unzipped the HC.zip. In the first one type

`java -jar HC_Gui.jar ENTER`,

in the second and third type

`java -jar HC_ClientAppl.jar ENTER`.

Now there should appear three windows, namely a control panel and two client panels, and a fourth one telling you „all clients are connected“ and asking you to start the game.

When you click OK, you will see a complete sequence of the HoneyComb© multi-client-game, but with only two participants. After this first rushing through, we will later go step by step through the demo. Click OK.

All the clicking for getting through the instructions and random moving over the playgrounds is generated by the program. You only can watch the process. You are seeing the screens for two players (clients) and the screen for the experimenter (server). The server as well as the clients are all running on localhost, the pseudo local area network on a single computer showing all windows on its single screen. Please arrange the overlapping windows on your screen to have them all in sight. The experimenter sees an overview of the clients, their positions coordinates on the hexgrid and the number of moves, each client already has made.

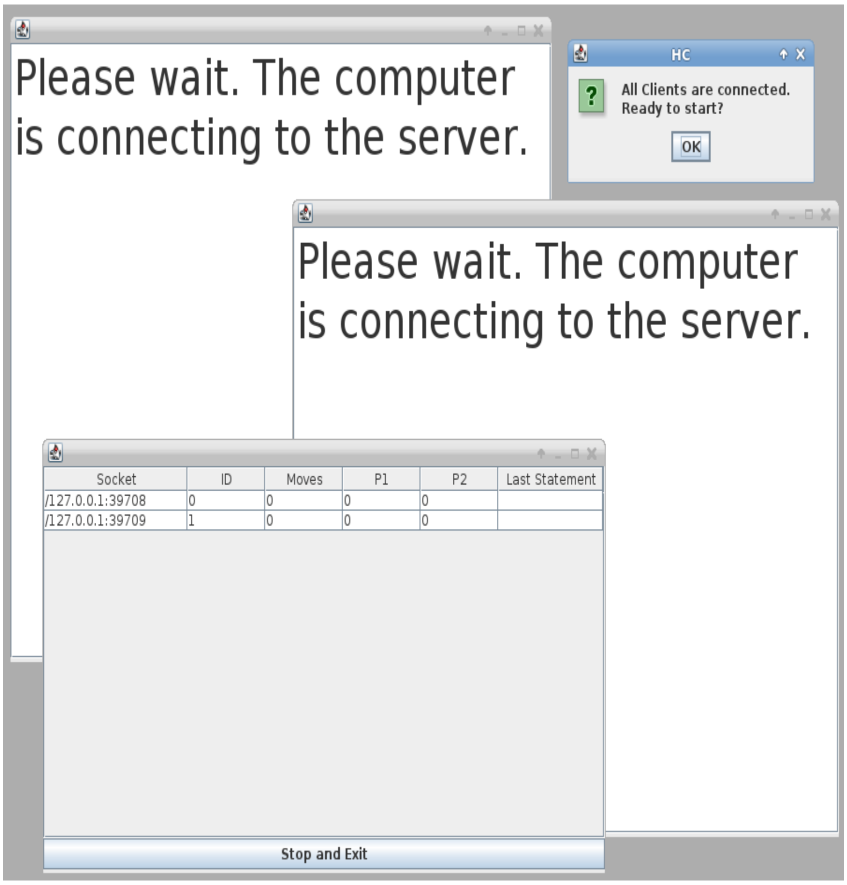

By the way, we had no problems to run the demo on the raspberry pi with raspbian as OS using several jvm's.

Leave the demo by pressing "Stop and exit". The control panel and all client windows will be closed by this action.

After this first rushing through, we will go step by step through the demo. Copy the file `hc_client.config` from the folder `demo-config-files` into the working directory. It differs from the default values in that `autoRead` and `autoMove` are set to false, so that the program will not generate clicks automatically any more, but you have to do that by yourself.

You can now start the game again by clicking on the `HC-demo.bat` (windows) or perform `HC-demo.sh` (Linux and Mac OS X).

Read the instructions to understand the sub games. Note, you are playing both players meaning that you will have to click through both instructions and make the moves of both players. In each sub game you have to make a minimal number of moves before the sub game is finished. In the demo-setting this number is reduced to only five moves per player.

Game 1 is just to learn how to move around on the playground by clicking into neighboring fields. In Game 2 you see your fictitious co-player and his/her moves on your panel, and she/he sees yours on her/his panel. Having done five moves in each panel you can proceed to two more preliminary games, dealing with picking up coins randomly hidden. If `testing` is still set to `true` in the `hc_client.config`, the doped fields as well as the fields you visited show their coordinates. This is not the case when `testing` is set to `false` in real experiments.

Sub games 1 and 2 are subject of the publication “Belz, M., Pyritz, L.W. & Boos, M. (2013). Spontaneous flocking in human groups. *Behavioural Processes, 92*, 6-14”.

Sub games 3 and 4 are subject of the manuscript “Belz, M. & Boos, M. (2013). Competitive outcome interdependence leads to reward-maximizing behavior, less pay satisfaction, and more stress. University of Göttingen: Unpublished Manuscript”.

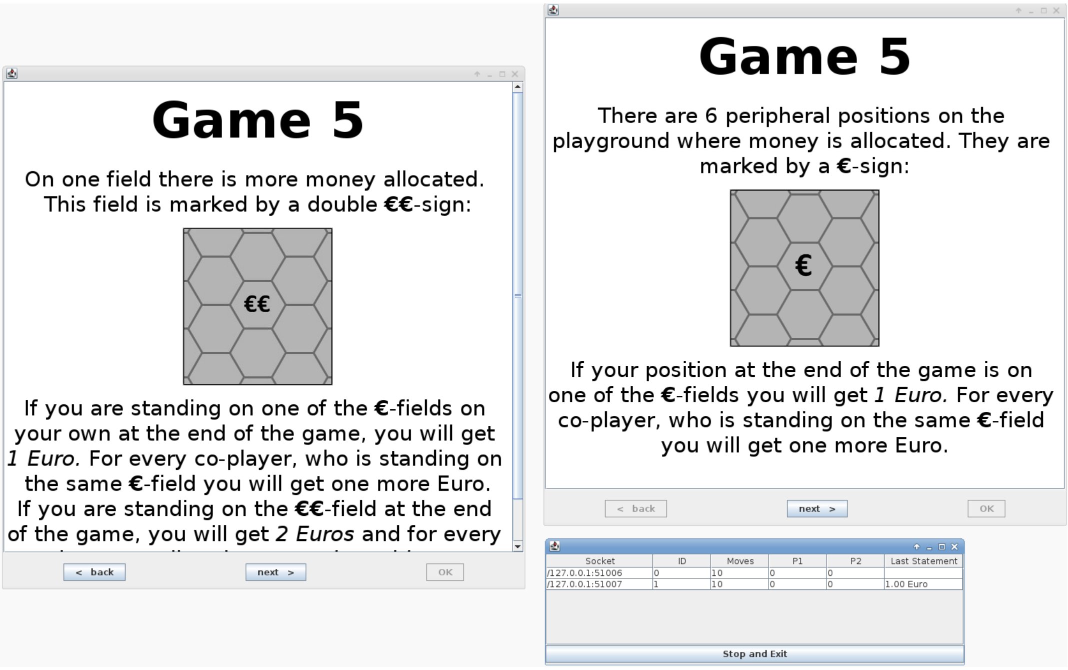

Please read the instructions of both players carefully when running the demo step by step to understand game 5. It is the main game, which is subject of this article „Boos, M., Pritz. J., Lange, S. & Belz, M. (2014). Leadership in moving human groups. PLOS Computational Biology“.

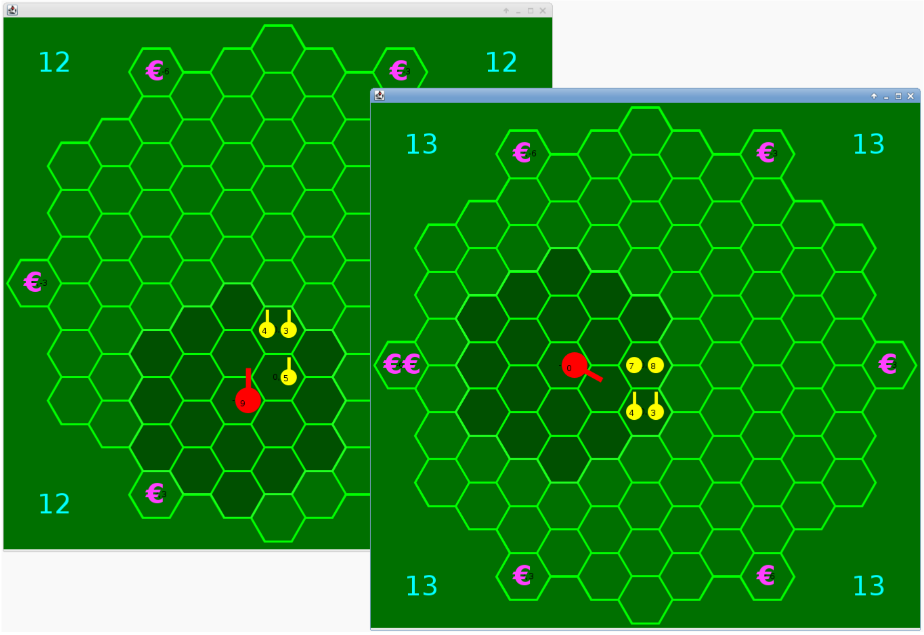

You can leave this demo by pressing "Stop and exit" on the sever-screen.

1.2 output

Now you have an idea how things work, and it's time to have a look into the output files, which can be found in the sub folder `rawdata`. Each pass of the game, the demo as well, leaves two output files in this folder, namely:

`HC_date_time.txt` and `HC_date_time_MOVES.csv`.

The first one contains starting times, positions and monetary gains. The second contains the pure moving data and some pseudo moves.

If you have run the demo twice you will find 4 files with today's date in their filenames.

Here is some explanation, how to interpret the content of the MOVES file:

headers of columns<br><br>`gnr; time ; pid; s1; s2;`	gnr: number of sub game<br>time: time stamp<br>pid player's id<br>coordinates in the hexgrid
`2; 10:32:20.060; -1; 0; 0;`	the pseudo pid -1 indicates the starting time,<br>here of sub game 2
`2; 10:32:20.060; 0; 0; 0;; not_moved`<br>`2; 10:32:20.060; 1; 0; 0;; not_moved`	starting positions of players with ids 0 and 1 in sub game 2
`4; 10:36:12.345; 1; -1; 2;;`<br>`4; 10:36:12.678; 0; -1; 2;;`<br>`. . .`<br>`. . .`	regular moves of player 2 in subgame 4 to field with coordinates (-1, 2) in the hexgrid (see the hexgrid in the appendix)

If you haven't run the demos, only the files

`HC_2010-08-04_14-05-11.txt`

`HC_2010-08-04_14-05-11_MOVES.csv`

will be in the folder named rawdata. These files are the original output belonging to the very same experimental group, which's screen-recorded and data-playbacked videos both are included in this article.

1.3 Configuring experiments

Let's have a closer look at the three config-files. You find them in the folder

`demo-config-files`, namely

`hc_client.config, hc_panel.config, hc_server.config`.

They go into effect when placed in the working directory from where the jars are started. Otherwise the program will use the built-in default configurations defined by us to give you a short overview of how the program looks like (see 1.1). Under Windows edit them with an editor like notepad++, which can handle Unix style line delimiters. Do not use Windows' built-in notepad.

To find out diffrent possible experimental setting play around with the numbers and values and see what happens. Each line in the config files ends with a hint, what it is operating. Or simply contact us at (HoneyComb@uni-goettingen.de) if you wish to use HoneyComb(c) for you own experiment.

1.3.1 hc_server.config

In the demo the number of players is only 2, in case of replicating our experiment it has to be 10.

1.3.2 hc_client.config

In the demo we set testing to true, to have the playgrounds in separate windows on a single screen. In the experimental setting we need fullscreen mode. We get it by setting

`testing = false`.

For the demo we used autoRead and autoMove to make the demo run automatically. Primarily these were used to test the HoneyComb© in a network. Setting `autoRead` and `autoMove` to `true` together with high values for numbers of moves in the `hc_server.config` allows hours of testing or demonstrating the software.

As a matter of fact the clients need the ip-number of the server to establish their connections.

The item `language` was added only now. Also the english set of instructions was added only now. The genuine experiments were performed with the German instructions. Keep this in mind when replicating the experiment with English speaking participants.

1.3.3 hc_panel.config

In the demo we enjoy a couple of bright colors, in the genuine experiment only grey shades were used. `testing = true` makes coordinates visible, when available.

The pair of `radius` (i.e. pixel radius) and `radiusPG` (i.e. radius of the playground measured in hexagons) is crucial, whether the whole playground fits onto the screen. For example the pair (40, 5) is suitable for the 1366x768 resolution of the notebooks we used in the genuine experiments as clients.

1.4 Instructions

The original German instruction files used in the genuine experiment are in the folder `intros/de`. Only now we added English translation in the folder `intros/en`. The instructions are given in simple html, as is interpreted by the java built-in interpreter. You can modify the texts, but do not change the names and the structure of the files. Be aware, that java interprets only a small subset of html. Anyhow we can use the intros.css and refer to images.

2 Replication

You find the original config files of the experiment, on which's data the article "Boos, M., Pritz. J., Lange, S. & Belz, M. (2014). Leadership in moving human groups. PLOS Computational Biology" is based, in the sub folder named

`replicate-config-files`.

To replicate the entire genuine setting you need a LAN with ten computers plus an eleventh which serves as the server, the others serve as clients.

Place **`HC_Gui.jar`** together with **`hc_server.config`** into a folder of the server.

Place **`HC_ClientAppl.jar`** together with **`hc_client.config`** and **`hc_hexpanel.config`** into a folder of each of the 10 client computers.

First, as ever, you start the server typing:

```
java -jar HC_Gui.jar,
```

afterwards start the ten clients, which get the server's IP via `hc_client.config`,

```
java -jar HC_ClientAppl.jar
```

You can perform the experiment in your local LAN as well as in other tcp/ip networks. A single WLAN, housing the server and all the clients is not appropriate.

Note: When a move is performed on a client computer, its new coordinates are sent to the server which updates its common playground and sends updated views of this to the clients. The client displays the updated playground not earlier than it receives the updated version from the server, even if it's the client, which caused the change by itself.

3 Playground

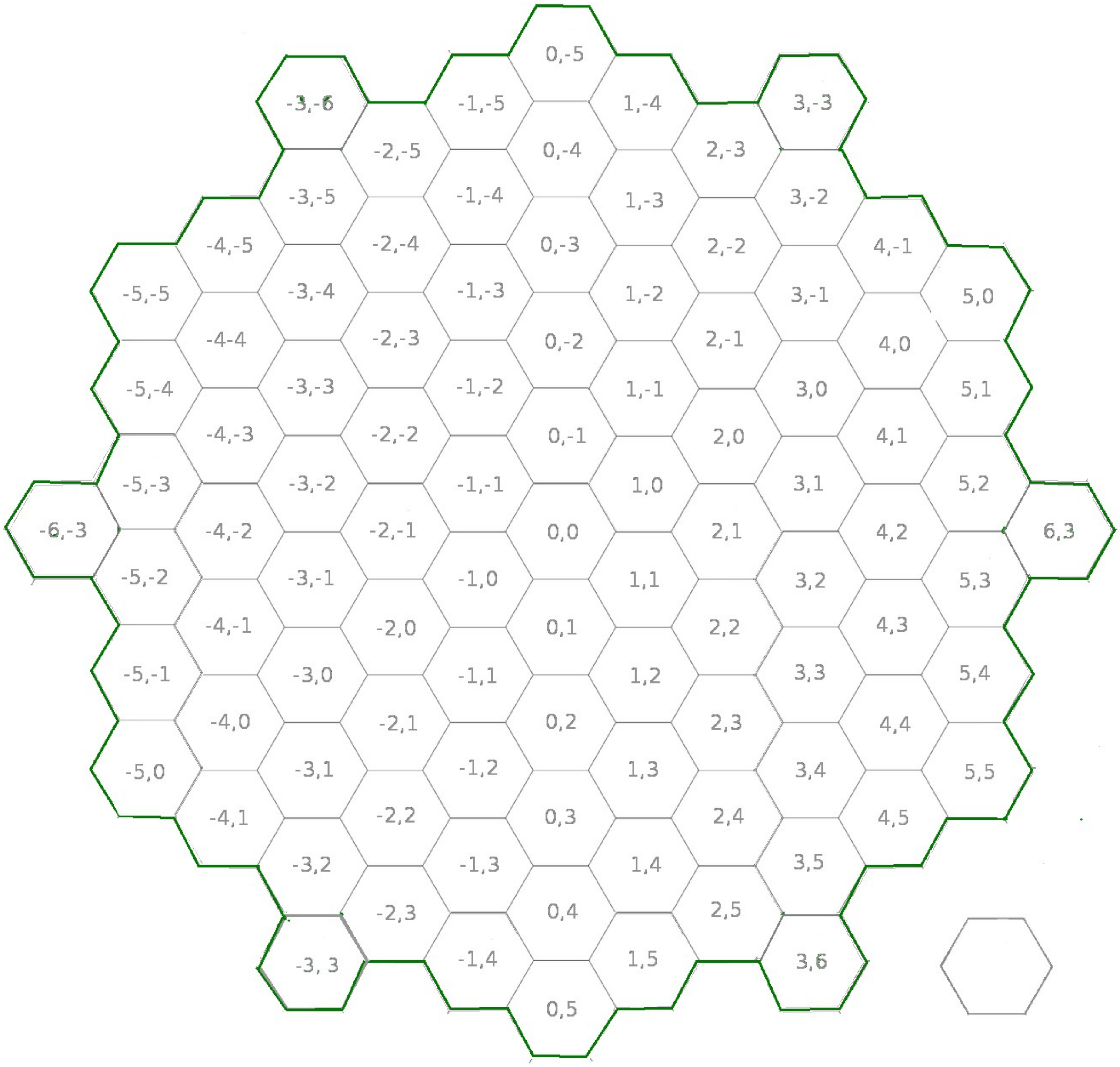

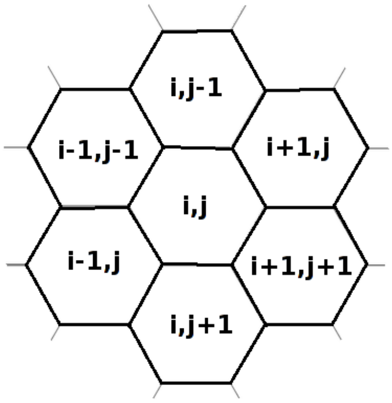

The playground consists of hexagons with coordinates as shown.

The HoneyComb(c) software (Jul 2010) and this guide (Jan 2014) are written by Johannes Pritz (jpritz@uni-goettingen.de).
