# Supplementary material for: Leadership in Moving Human Groups
Source: PLoS Comput Biol. 2014 Apr 3;10(4):e1003541. doi: 10.1371/journal.pcbi.1003541 (PMC3974633; doi:10.1371/journal.pcbi.1003541)
Supplement: Software S1 — Archive version of the software which was used for the experiment. (ZIP) [file pcbi.1003541.s002.zip › intro/de/HC_Willkommen.html]

Willkommen


# Willkommen!

  
  

  

Sie werden in wenigen Minuten zusammen mit Ihren Mitspielerinnen und
Mitspielern an einem Spiel um echtes Geld teilnehmen.   
   
Bitte klicken Sie unten auf den Weiter-Button, um zu beginnen.
