# Supplementary material for: Leadership in Moving Human Groups
Source: PLoS Comput Biol. 2014 Apr 3;10(4):e1003541. doi: 10.1371/journal.pcbi.1003541 (PMC3974633; doi:10.1371/journal.pcbi.1003541)
Supplement: Software S1 — Archive version of the software which was used for the experiment. (ZIP) [file pcbi.1003541.s002.zip › intro/de/HC_spiel1_2.html]

Erste Übung global


# Spiel 1

Sie können einen Zug ausführen, indem Sie mit der Maus
direkt in ein benachbartes Feld klicken. Das Überspringen von
Feldern ist nicht möglich.
