# Supplementary material for: Leadership in Moving Human Groups
Source: PLoS Comput Biol. 2014 Apr 3;10(4):e1003541. doi: 10.1371/journal.pcbi.1003541 (PMC3974633; doi:10.1371/journal.pcbi.1003541)
Supplement: Software S1 — Archive version of the software which was used for the experiment. (ZIP) [file pcbi.1003541.s002.zip › intro/de/HC_spiel1_3.html]

Erste Übung global


# Spiel 1

Sie werden bemerken, dass an Ihrer Figur nach jedem Zug für
eine kurze Zeitspanne eine Linie eingeblendet wird. Diese Linie zeigt
Ihnen die Richtung an, in die Sie sich zuletzt bewegt haben:

Nach einem kurzen Augenblick wird die Linie ausgeblendet. Sie
können sich mit dem nächsten Zug so viel Zeit lassen, wie
Sie möchten.
