# Supplementary material for: Leadership in Moving Human Groups
Source: PLoS Comput Biol. 2014 Apr 3;10(4):e1003541. doi: 10.1371/journal.pcbi.1003541 (PMC3974633; doi:10.1371/journal.pcbi.1003541)
Supplement: Software S1 — Archive version of the software which was used for the experiment. (ZIP) [file pcbi.1003541.s002.zip › intro/de/HC_spiel1_4.html]

Erste Übung global


# Spiel 1

Sie können nicht unmittelbar hintereinander zwei Züge
ausführen. Wenn Sie einen Zug gemacht haben, wird sich Ihr
Mauszeiger für einige Sekunden in eine Sanduhr verwandeln.
Während dieser Zeitspanne ist es Ihnen nicht möglich, weiter
zu ziehen.

Das erste Spiel ist beendet, wenn Sie **mindestens 15 Züge**
gemacht haben.   
 Bitte klicken Sie unten auf den OK-Button, um
mit dem Spiel zu beginnen. Wenn Sie an dieser Stelle oder während
des Spiels Verständnisfragen haben, wenden Sie sich bitte an den
Versuchsleiter.
