# Supplementary material for: Leadership in Moving Human Groups
Source: PLoS Comput Biol. 2014 Apr 3;10(4):e1003541. doi: 10.1371/journal.pcbi.1003541 (PMC3974633; doi:10.1371/journal.pcbi.1003541)
Supplement: Software S1 — Archive version of the software which was used for the experiment. (ZIP) [file pcbi.1003541.s002.zip › intro/de/HC_spiel1_lokal1.html]

Erste Übung global


# Spiel 1

Bitte lesen Sie die folgenden Informationen gut durch und klicken Sie
anschließend auf "weiter". Sie haben immer die
Möglichkeit, durch Klicken auf "zurück" die
jeweils vorige Seite anzeigen zu lassen.   
  
 In diesem ersten Spiel werden Sie gleich ein Spielfeld aus
Sechsecken sehen. Sie repräsentieren den Punkt in der Mitte des
Spielfeldes. Die schwarze Linie, die mehrere Sechsecke umrandet,
stellt Ihren Sichtradius dar:
