# Supplementary material for: Leadership in Moving Human Groups
Source: PLoS Comput Biol. 2014 Apr 3;10(4):e1003541. doi: 10.1371/journal.pcbi.1003541 (PMC3974633; doi:10.1371/journal.pcbi.1003541)
Supplement: Software S1 — Archive version of the software which was used for the experiment. (ZIP) [file pcbi.1003541.s002.zip › intro/de/HC_spiel2_1.html]

Zweite Übung global


# Spiel 2

Bitte lesen Sie die folgenden Informationen gut durch und klicken sie
anschließend auf "weiter". Sie haben immer die
Möglichkeit, durch Klicken auf "zurück" die
jeweils vorige Seite anzeigen zu lassen.   
   
 In diesem
zweiten Spiel können Sie Ihren Punkt genau so bewegen wie im
ersten Spiel vorher. Im Gegensatz zum ersten Spiel werden Sie Ihre
Mitspielerinnen und Mitspieler nun jedoch ebenfalls als kleine Punkte
auf dem Spielfeld sehen können. Sie selbst sind der
größte Punkt:
