# Supplementary material for: Leadership in Moving Human Groups
Source: PLoS Comput Biol. 2014 Apr 3;10(4):e1003541. doi: 10.1371/journal.pcbi.1003541 (PMC3974633; doi:10.1371/journal.pcbi.1003541)
Supplement: Software S1 — Archive version of the software which was used for the experiment. (ZIP) [file pcbi.1003541.s002.zip › intro/de/HC_spiel2_lokal2.html]

Zweite Übung global


# Spiel 2

Nach jedem Zug werden Hilfslinien eingeblendet, die Ihnen
verdeutlichen, in welche Richtung sich Ihre Mitspielerinnen und
Mitspieler und auch Sie selbst bewegt haben. Wie im ersten Spiel
verschwinden diese Linien, falls Sie oder Ihre Mitspielerinnen und
Mitspieler eine Weile keinen Zug ausführen. Bitte denken Sie
daran, dass Sie nur Mitspielerinnen und Mitspieler sehen können,
die sich in Ihrem Sichtradius (schwarze Umrandung) befinden.

Das zweite Spiel ist beendet, wenn Sie **mindesten 15 Züge**
gemacht haben.   
 Bitte klicken Sie unten auf den OK-Button, um
mit dem Spiel zu beginnen. Wenn Sie an dieser Stelle oder während
des Spiels Verständnisfragen haben, wenden Sie sich bitte an den
Versuchsleiter.
