# Supplementary material for: Leadership in Moving Human Groups
Source: PLoS Comput Biol. 2014 Apr 3;10(4):e1003541. doi: 10.1371/journal.pcbi.1003541 (PMC3974633; doi:10.1371/journal.pcbi.1003541)
Supplement: Software S1 — Archive version of the software which was used for the experiment. (ZIP) [file pcbi.1003541.s002.zip › intro/de/HC_spiel3_1.html]

Experiment Phase 1


# Spiel 3

Auf dem Spielfeld sind an zufälligen Stellen einige unsichtbare
*0,50 Euro-Stücke* versteckt, die Sie und Ihre Mitspielerinnen und
Mitspieler finden können. Wenn Sie ein Geldstück auf einem
Feld entdeckt haben, gehört es unwiderruflich Ihnen. Es erscheint
dann auf dem Feld die folgende, **nur für Sie sichtbare**
Markierung:

Jedes einzelne 0,50 Euro-Stück kann **pro Person einmal
gefunden werden**. Entdeckt eine Mitspielerin oder ein Mitspieler ein
0,50 Euro-Stück vor Ihnen, hat dies also **keine Konsequenz**;
das Geld bleibt an Ort und Stelle und kann immer noch von Ihnen
entdeckt werden.
