# Supplementary material for: Leadership in Moving Human Groups
Source: PLoS Comput Biol. 2014 Apr 3;10(4):e1003541. doi: 10.1371/journal.pcbi.1003541 (PMC3974633; doi:10.1371/journal.pcbi.1003541)
Supplement: Software S1 — Archive version of the software which was used for the experiment. (ZIP) [file pcbi.1003541.s002.zip › intro/de/HC_spiel4_1.html]

Experiment Phase 1


# Spiel 4

Auf dem Spielfeld sind an zufälligen Stellen einige unsichtbare *0,50
Euro-Stücke* versteckt, die Sie oder Ihre Mitspielerinnen und
Mitspieler finden können. Wenn Sie ein Geldstück auf einem
Feld entdeckt haben, gehört es unwiderruflich Ihnen. Es erscheint
dann auf dem Feld die folgende, **für alle sichtbare**
Markierung:

Jedes 0,50 Euro-Stück kann **nur ein einziges Mal gefunden
werden**. Wenn Sie oder eine Mitspielerin bzw. ein Mitspieler ein 0,50
Euro-Stück finden, **wird es aus dem Spiel genommen und ist
für niemanden mehr auffindbar**.
