# Supplementary material for: Leadership in Moving Human Groups
Source: PLoS Comput Biol. 2014 Apr 3;10(4):e1003541. doi: 10.1371/journal.pcbi.1003541 (PMC3974633; doi:10.1371/journal.pcbi.1003541)
Supplement: Software S1 — Archive version of the software which was used for the experiment. (ZIP) [file pcbi.1003541.s002.zip › intro/de/HC_spiel4_2.html]

# Spiel 4

Bitte denken Sie daran, dass Sie **maximal 15 Züge** machen
können. Die verbleibenden Züge werden Ihnen in den vier
Ecken rund um das Spielfeld angezeigt. In diesem Beispiel haben Sie
noch 14 Züge übrig:

Das Spiel ist beendet, wenn alle Spielerinnen und Spieler ihre
Züge aufgebraucht haben. Bitte klicken Sie unten auf den
OK-Button, um mit dem Spiel zu beginnen.
