# Supplementary material for: Leadership in Moving Human Groups
Source: PLoS Comput Biol. 2014 Apr 3;10(4):e1003541. doi: 10.1371/journal.pcbi.1003541 (PMC3974633; doi:10.1371/journal.pcbi.1003541)
Supplement: Software S1 — Archive version of the software which was used for the experiment. (ZIP) [file pcbi.1003541.s002.zip › intro/de/HC_spiel5_inf1.html]

Experiment informiert


# Spiel 5

Es gibt auf dem Spielfeld insgesamt 6 Geld-Depots, deren Positionen
gleich auf Ihrer Karte zu sehen sein werden. 5 dieser 6 Geld-Depots
werden durch ein **€**-Zeichen markiert:
