# Supplementary material for: Leadership in Moving Human Groups
Source: PLoS Comput Biol. 2014 Apr 3;10(4):e1003541. doi: 10.1371/journal.pcbi.1003541 (PMC3974633; doi:10.1371/journal.pcbi.1003541)
Supplement: Software S1 — Archive version of the software which was used for the experiment. (ZIP) [file pcbi.1003541.s002.zip › intro/de/HC_spiel5_inf7.html]

Experiment uninformiert


# Spiel 5

Bitte denken Sie daran, dass Sie nur **maximal 15 Züge**
machen können. Wenn Sie am Ende des Spiels nicht auf einem
Geld-Depot stehen, erhalten Sie *kein Geld*. Die verbleibenden
Züge werden Ihnen in den vier Ecken rund um das Spielfeld
angezeigt. In diesem Beispiel haben Sie noch 14 Züge übrig:
