# Supplementary material for: Leadership in Moving Human Groups
Source: PLoS Comput Biol. 2014 Apr 3;10(4):e1003541. doi: 10.1371/journal.pcbi.1003541 (PMC3974633; doi:10.1371/journal.pcbi.1003541)
Supplement: Software S1 — Archive version of the software which was used for the experiment. (ZIP) [file pcbi.1003541.s002.zip › intro/de/HC_spiel5_inf8.html]

Experiment uninformiert


# Spiel 5

Das Spiel wird beendet, sobald alle Spielerinnen und Spieler auf
Geld-Depots stehen oder alle Züge sämtlicher Spielerinnen
und Spieler aufgebraucht sind. **Sie müssen also nicht
zwingend alle Ihre Züge aufbrauchen.**   
   
 Bitte
klicken Sie unten auf den OK-Button, um mit dem Spiel zu beginnen.
