# Supplementary material for: Leadership in Moving Human Groups
Source: PLoS Comput Biol. 2014 Apr 3;10(4):e1003541. doi: 10.1371/journal.pcbi.1003541 (PMC3974633; doi:10.1371/journal.pcbi.1003541)
Supplement: Software S1 — Archive version of the software which was used for the experiment. (ZIP) [file pcbi.1003541.s002.zip › intro/de/HC_spiel5_uninf1.html]

Experiment uninformiert


# Spiel 5

Es gibt auf dem Spielfeld insgesamt 6 Geld-Depots, deren Positionen
gleich auf Ihrer Karte zu sehen sein werden. Alle Geld-Depots werden
durch ein **€**-Zeichen markiert:

Wenn Sie am Ende des Spiels alleine auf einem dieser **€**-Felder
stehen, erhalten Sie *1 Euro*. Für jede Mitspielerin und
jeden Mitspieler, die/der am Ende *zusammen mit Ihnen* auf dem **€**-Feld
steht, erhalten Sie *1 Euro zusätzlich.*
