# Supplementary material for: Leadership in Moving Human Groups
Source: PLoS Comput Biol. 2014 Apr 3;10(4):e1003541. doi: 10.1371/journal.pcbi.1003541 (PMC3974633; doi:10.1371/journal.pcbi.1003541)
Supplement: Software S1 — Archive version of the software which was used for the experiment. (ZIP) [file pcbi.1003541.s002.zip › intro/en/HC_spiel1_3.html]

First Exercise Global


# Game 1

You will observe that your figure will have a tail after each
move for some seconds. This tail points into the direction, you are
coming from.

After some seconds the tail disappears. You can take your time
as you like until your next move.
