# Supplementary material for: Leadership in Moving Human Groups
Source: PLoS Comput Biol. 2014 Apr 3;10(4):e1003541. doi: 10.1371/journal.pcbi.1003541 (PMC3974633; doi:10.1371/journal.pcbi.1003541)
Supplement: Software S1 — Archive version of the software which was used for the experiment. (ZIP) [file pcbi.1003541.s002.zip › intro/en/HC_spiel1_4.html]

First Exercise Global


# Game 1

When you have performed a move, you can not immediatly make the
next one. Your mouse pointer will change to an hourglass for a few
seconds, during this period you are not able to perform an other move.

The first game is finished when you have made **at least 15 moves**.
  
 Please click the OK-Button, to start the game. If you have
any questions now or later during the game please ask the
experimenter.
