# Supplementary material for: Leadership in Moving Human Groups
Source: PLoS Comput Biol. 2014 Apr 3;10(4):e1003541. doi: 10.1371/journal.pcbi.1003541 (PMC3974633; doi:10.1371/journal.pcbi.1003541)
Supplement: Software S1 — Archive version of the software which was used for the experiment. (ZIP) [file pcbi.1003541.s002.zip › intro/en/HC_spiel1_lokal1.html]

First


# Game 1

Please read the following information carefully and click on
"next" afterwards. You can always go back by clicking
"back" to see previous pages again.   
  
 In this first game you will see a playground consisting of
hexagons. You are represented by a dot in the hexagon in the middle of
the playground.   
 The black line surrounding your neighboring
fields indicates your visual range.
