# Supplementary material for: Leadership in Moving Human Groups
Source: PLoS Comput Biol. 2014 Apr 3;10(4):e1003541. doi: 10.1371/journal.pcbi.1003541 (PMC3974633; doi:10.1371/journal.pcbi.1003541)
Supplement: Software S1 — Archive version of the software which was used for the experiment. (ZIP) [file pcbi.1003541.s002.zip › intro/en/HC_spiel1_lokal2.html]

Erste Übung global


# Game 1

You can perform a move by clicking directly in one of the
neighboring fields. Jumping over fields is not possible.
