# Supplementary material for: Leadership in Moving Human Groups
Source: PLoS Comput Biol. 2014 Apr 3;10(4):e1003541. doi: 10.1371/journal.pcbi.1003541 (PMC3974633; doi:10.1371/journal.pcbi.1003541)
Supplement: Software S1 — Archive version of the software which was used for the experiment. (ZIP) [file pcbi.1003541.s002.zip › intro/en/HC_spiel1_lokal4.html]

First Exercise Local


# Game 1

When you have performed a move, you cannot immediatly make the
next one. When you have performed one, your mouse pointer will change
into an hourglass for some seconds. During this period you are not
able to perform another move.

The first game is finished when you have made **at least 15 moves**.
  
 Please click the OK-Button, to start the game. If you have
any questions now or later during the game please ask the
experimenter.
