# Supplementary material for: Leadership in Moving Human Groups
Source: PLoS Comput Biol. 2014 Apr 3;10(4):e1003541. doi: 10.1371/journal.pcbi.1003541 (PMC3974633; doi:10.1371/journal.pcbi.1003541)
Supplement: Software S1 — Archive version of the software which was used for the experiment. (ZIP) [file pcbi.1003541.s002.zip › intro/en/HC_spiel2_1.html]

Second Exercise Global


# Game 2

Please read the following information carefully and click on
"next" afterwards. You can always go back by clicking
"back" to see previous pages again.   
  
 In this second game you can move your dot in the same way as
in the first game, but now you can see your co-player being small dots
as well. Your dot is the biggest one.
