# Supplementary material for: Leadership in Moving Human Groups
Source: PLoS Comput Biol. 2014 Apr 3;10(4):e1003541. doi: 10.1371/journal.pcbi.1003541 (PMC3974633; doi:10.1371/journal.pcbi.1003541)
Supplement: Software S1 — Archive version of the software which was used for the experiment. (ZIP) [file pcbi.1003541.s002.zip › intro/en/HC_spiel2_lokal2.html]

Second Exercise Global


# Game 2

After each move your dot will have a little tail indicating the
direction which you are coming from. The same applies to your
co-players dots. As in the first game, these tails disappear after a
while without movement.  
 Please keep in mind that you can see
only those co-players who are within your visibility range, wich is
indicated by a black boundary.

The second game is finished as soon as you have performed at least 15
moves.   
 Please click the OK-Button to start the game. If you
have any questions ask the experimenter.
