# Supplementary material for: Leadership in Moving Human Groups
Source: PLoS Comput Biol. 2014 Apr 3;10(4):e1003541. doi: 10.1371/journal.pcbi.1003541 (PMC3974633; doi:10.1371/journal.pcbi.1003541)
Supplement: Software S1 — Archive version of the software which was used for the experiment. (ZIP) [file pcbi.1003541.s002.zip › intro/en/HC_spiel3_1.html]

Experimental Phase 1


# Game 3

A number of 0.50 Euro coins are hidden in random places on the
playground. If you enter such a field, the field will be marked and
the coin is yours. Note, the mark is only visible for you and you
cannot see marks of fields, where your co-players have found coins.

When you enter a coin field you will receive the coin, even if
someone else has entered the same field before and already got a coin
there for himself/herself.
