# Supplementary material for: Leadership in Moving Human Groups
Source: PLoS Comput Biol. 2014 Apr 3;10(4):e1003541. doi: 10.1371/journal.pcbi.1003541 (PMC3974633; doi:10.1371/journal.pcbi.1003541)
Supplement: Software S1 — Archive version of the software which was used for the experiment. (ZIP) [file pcbi.1003541.s002.zip › intro/en/HC_spiel3_2.html]

Experimental Phase 1


# Game 3

Please keep in mind that you can make **15 moves at most**. The
number of remaining moves is shown in the four corners around the
playground. In the example shown below you have still got 14 moves
left:

The game will be over when everybody has used up all his moves.  
Please click OK to start the game.
