# Supplementary material for: Leadership in Moving Human Groups
Source: PLoS Comput Biol. 2014 Apr 3;10(4):e1003541. doi: 10.1371/journal.pcbi.1003541 (PMC3974633; doi:10.1371/journal.pcbi.1003541)
Supplement: Software S1 — Archive version of the software which was used for the experiment. (ZIP) [file pcbi.1003541.s002.zip › intro/en/HC_spiel4_1.html]

Experiment Phase 2


# Game 4

There are a number of 0.50 Euro coins, hidden in random places
on the playground. If you or someone else enters such a field the
field will be marked and the one who has entered the field first gets
the coin. Note, the mark is visible for everybody, where a coin is
taken away no further coin can be picked up.

So every 0.50 Euro coin can **only be found once**.
