# Supplementary material for: Leadership in Moving Human Groups
Source: PLoS Comput Biol. 2014 Apr 3;10(4):e1003541. doi: 10.1371/journal.pcbi.1003541 (PMC3974633; doi:10.1371/journal.pcbi.1003541)
Supplement: Software S1 — Archive version of the software which was used for the experiment. (ZIP) [file pcbi.1003541.s002.zip › intro/en/HC_spiel5_inf2.html]

Experiment informed


# Game 5

On one field there is more money allocated. This field is marked by a
double **€€**-sign:

If you are standing on one of the **€**-fields on your own at
the end of the game, you will get *1 Euro.* For every co-player,
who is standing on the same **€**-field you will get one more
Euro.   
 If you are standing on the **€€**-field
at the end of the game, you will get *2 Euros* and for every
co-player standing there *together with you* on the **€€**-field
you will get *2 more Euros.*
