# Supplementary material for: Leadership in Moving Human Groups
Source: PLoS Comput Biol. 2014 Apr 3;10(4):e1003541. doi: 10.1371/journal.pcbi.1003541 (PMC3974633; doi:10.1371/journal.pcbi.1003541)
Supplement: Software S1 — Archive version of the software which was used for the experiment. (ZIP) [file pcbi.1003541.s002.zip › intro/en/HC_spiel5_inf6.html]

Experiment uinformed


# Game 5

Example 4: At the end of the game you are standing together
with 4 co-player on the **€€**-field. You will get *10 Euros.*
