# Supplementary material for: Leadership in Moving Human Groups
Source: PLoS Comput Biol. 2014 Apr 3;10(4):e1003541. doi: 10.1371/journal.pcbi.1003541 (PMC3974633; doi:10.1371/journal.pcbi.1003541)
Supplement: Software S1 — Archive version of the software which was used for the experiment. (ZIP) [file pcbi.1003541.s002.zip › intro/en/HC_spiel5_inf7.html]

Experiment informed


# Game 5

Please keep in mind that you can make **15 moves at most**. If you
are not standing on a money-field in the end of the game, you will not
get any money at all.   
 At any time your remaining moves are
shown in the four corners around the playground. In the example below
you have got 14 moves left:
