# Supplementary material for: Leadership in Moving Human Groups
Source: PLoS Comput Biol. 2014 Apr 3;10(4):e1003541. doi: 10.1371/journal.pcbi.1003541 (PMC3974633; doi:10.1371/journal.pcbi.1003541)
Supplement: Software S1 — Archive version of the software which was used for the experiment. (ZIP) [file pcbi.1003541.s002.zip › intro/en/HC_spiel5_uninf1.html]

Experiment uninformed


# Game 5

There are 6 peripheral positions on the playground where money is
allocated. They are marked by a **€**-sign:

If your position at the end of the game is on one of the **€**-fields
you will get *1 Euro.* For every co-player, who is standing on
the same **€**-field you will get one more Euro.
