# Supplementary material for: Leadership in Moving Human Groups
Source: PLoS Comput Biol. 2014 Apr 3;10(4):e1003541. doi: 10.1371/journal.pcbi.1003541 (PMC3974633; doi:10.1371/journal.pcbi.1003541)
Supplement: Software S1 — Archive version of the software which was used for the experiment. (ZIP) [file pcbi.1003541.s002.zip › intro/en/HC_spiel5_uninf2.html]

Experiment uninformed


# Game 5

Example 1: At the end of the game only you are standing on a **€**-Feld,
but no one else is standing on this field. You will get *1 Euro.*
