# Supplementary material for: Leadership in Moving Human Groups
Source: PLoS Comput Biol. 2014 Apr 3;10(4):e1003541. doi: 10.1371/journal.pcbi.1003541 (PMC3974633; doi:10.1371/journal.pcbi.1003541)
Supplement: Software S1 — Archive version of the software which was used for the experiment. (ZIP) [file pcbi.1003541.s002.zip › intro/en/HC_spiel5_uninf4.html]

Experiment uninformed


# Game 5

Please keep in mind that you can make **15 moves at most**. If you
are not standing on a money-field at the end of the game, you will get
*no money* at all.   
 The number of remaining moves is
shown in the four corners around the playground. In the example below
you have got 14 moves left:
