# Supplementary material for: Leadership in Moving Human Groups
Source: PLoS Comput Biol. 2014 Apr 3;10(4):e1003541. doi: 10.1371/journal.pcbi.1003541 (PMC3974633; doi:10.1371/journal.pcbi.1003541)
Supplement: Software S1 — Archive version of the software which was used for the experiment. (ZIP) [file pcbi.1003541.s002.zip › intro/en/HC_spiel5_uninf5.html]

Experiment uninformiert


# Game 5

The game is finished, as soon as all co-players are standing on
money-fields and/or those not standing on a money-field have got no
moves left. Therefore there is no need to use up your moves.   
  
 Please click the OK-Button to start the game.
