# Supplementary material for: Leadership in Moving Human Groups
Source: PLoS Comput Biol. 2014 Apr 3;10(4):e1003541. doi: 10.1371/journal.pcbi.1003541 (PMC3974633; doi:10.1371/journal.pcbi.1003541)
Supplement: Text S1 — Legend to Video S1. (DOCX) [file pcbi.1003541.s006.docx]

**Example of collective movement from the perspective of an uninformed player**

For visualisation purposes, we include two video files with movies from one experimental group. Video S1 is recorded from the screen during an experimental session, including cursor movement. It shows the perspective of an uninformed player with local visual perception who reaches one ‘€’ depot at the bottom right as last player of the group.
