# Supplementary material for: Leadership in Moving Human Groups
Source: PLoS Comput Biol. 2014 Apr 3;10(4):e1003541. doi: 10.1371/journal.pcbi.1003541 (PMC3974633; doi:10.1371/journal.pcbi.1003541)
Supplement: Text S2 — Legend to Video S2. (DOCX) [file pcbi.1003541.s007.docx]

**Example of collective movement from the same group – from perspectives of an uninformed and an informed player**

Video S2 is a playback fed with the data from the same session as Video S1. It shows two perspectives within the same time frame: (a) on the right side, the perspective of the uninformed player from Video S1, (b) on the left side, the perspective of one informed player, who can see one ‘€€’ money depot in place of the ‘€’ money depot at the bottom right.
